# Supplementary material for: Vision impairment and associated daily activity limitation: A systematic review and meta-analysis
Source: PLoS One. 2025 Jan 31;20(1):e0317452. doi: 10.1371/journal.pone.0317452 (PMC11785307; doi:10.1371/journal.pone.0317452)
Supplement: S1 Table — (DOCX) [file pone.0317452.s003.docx]

# **Supplementary Table 1.** Literature search strategy

**Text 1. MEDLINE**

1. exp eye diseases/

2. Visually Impaired Persons/

3. ((low$ or handicap$ or subnormal$ or impair$ or partial$ or disab$ or disorder$ or loss$ or limit$) adj3 (vision or visual$ or sight$)).tw.

4. ((macul$ or retina$ or choroid$) adj3 degener$).tw.

5. ((macul$ or retina$ or choroid$) adj3 neovasc$).tw.

6. (AMD or ARMD).tw.

7. ((diabet$ or proliferative or non-proliferative or pre-proliferative) adj4 retinopath$).tw.

8. (diabet$ adj2 (eye$ or vision or visual$ or sight$)).tw.

9. glaucoma$.tw.

10. cataract$.tw.

11. blindness.tw.

12. Visual Acuity/

13. visual acuit$.tw.

14. Contrast Sensitivity/

15. (contrast adj2 sensitivity).tw.

16. Depth Perception/

17. stereopsis.tw.

18. (stereo adj1 acuit$).tw.

19. Visual Fields/

20. ((visual$ or vision) adj2 function$).tw.

21. or/1-20

22. exp Activity of Daily Living /

23. Instrumental Activity of Daily Living /

24. activity of daily living.tw.

25. instrumental activity of daily living.tw

26. (ADL or IADL or bathing or dressing or toileting or transfer or chair or beds or continence).tw.

27. or/22-26

28. Cohort Studies/

29. Longitudinal Studies/

30. (cohort$ or longitudinal).tw.

31. Cross-Sectional Studies/

32. "Surveys and Questionnaires"/

33. Health Surveys/

34. (survey or surveys).tw.

35. or/28-34

36. 21 and 27 and 35

­

**Text 2. Embase**

1. exp eye disease/co, cn, di, dm, dr, dt, ep, et, pc, rt, rh, su, th [Complication, Congenital Disorder, Diagnosis, Disease Management, Drug

Resistance, Drug Therapy, Epidemiology, Etiology, Prevention, Radiotherapy, Rehabilitation, Surgery, Therapy]

2. exp visual impairment/

3. ((low$ or handicap$ or subnormal$ or impair$ or partial$ or disab$ or disorder$ or loss$ or limit$) adj3 (vision or visual$ or sight$)).tw.

4. ((macul$ or retina$ or choroid$) adj3 degener$).tw.

5. ((macul$ or retina$ or choroid$) adj3 neovasc$).tw.

6. (AMD or ARMD).tw.

7. ((diabet$ or proliferative or non-proliferative or pre-proliferative) adj4 retinopath$).tw.

8. (diabet$ adj2 (eye$ or vision or visual$ or sight$)).tw.

9. glaucoma$.tw.

10. cataract$.tw.

11. blindness.tw.

12. visual acuity/

13. visual acuit$.tw.

14. contrast sensitivity/

15. (contrast adj2 sensitivity).tw.

16. stereoscopic vision/

17. stereopsis.tw.

18. (stereo adj1 acuit$).tw.

19. visual field defect/

20. visual field/

21. ((visual$ or vision) adj2 function$).tw.

22. or/1-21

23. exp Activity of Daily Living /

24. Instrumental Activity of Daily Living /

25. activity of daily living.tw.

26. instrumental activity of daily living.tw.

27. (ADL or IADL or bathing or dressing or toileting or transfer or chair or beds or continence).tw.

28. or/23-27

29. cohort analysis/

30. longitudinal study/

31. (cohort$ or longitudinal).tw.

32. cross-sectional study/

33. questionnaire/

34. health survey/ or health care survey/

35. (survey or surveys).tw.

36. or/29-35

37. 22 and 28 and 36

**Text 2. CENTRAL**

1. eye diseases/

2. vision/

3. vision disorders/

4. ((low$ or handicap$ or subnormal$ or impair$ or partial$ or disab$ or disorder$ or loss$ or limit$) adj3 (vision or visual$ or sight$)).tw.

5. ((macul$ or retina$ or choroid$) adj3 degener$).tw.

6. ((macul$ or retina$ or choroid$) adj3 neovasc$).tw.

7. (AMD or ARMD).tw.

8. ((diabet$ or proliferative or non-proliferative or pre-proliferative) adj4 retinopath$).tw.

9. (diabet$ adj2 (eye$ or vision or visual$ or sight$)).tw.

10. glaucoma$.tw.

11. cataract$.tw.

12. blindness.tw.

13. visual acuit$.tw.

14. (contrast adj2 sensitivity).tw.

15. stereopsis.tw.

16. (stereo adj1 acuit$).tw.

17. ((visual$ or vision) adj2 function$).tw.

18. or/1-17

19. Activity of Daily Living /

20. Instrumental Activity of Daily Living /

21. activity of daily living.tw.

22. instrumental activity of daily living.tw.

23. (ADL or IADL or bathing or dressing or toileting or transfer or chair or beds or continence).tw.

24. or/19-23

25. cohort studies/

26. longitudinal studies/

27. (cohort$ or longitudinal).tw.

28. questionnaires/

29. surveys/

30. (survey or surveys).tw.

31. or/25-30

32. 18 and 24 and 31
