# Supplementary material for: Vision impairment and associated daily activity limitation: A systematic review and meta-analysis
Source: PLoS One. 2025 Jan 31;20(1):e0317452. doi: 10.1371/journal.pone.0317452 (PMC11785307; doi:10.1371/journal.pone.0317452)
Supplement: S3 Table — (DOCX) [file pone.0317452.s005.docx]

# **Supplementary Table 3.** A list of the excluded studies and reasons for their exclusion

|  | **Studies** | **Exclusion Reason** |
| --- | --- | --- |
| 1 | Azoulay-Sebban L, Zhao Z, Zenouda A, Lombardi M, Gutman E, Brasnu E, Hamard P, Sahel JA, Baudouin C, Labbé A. Correlations between subjective evaluation of quality of life, visual field loss, and performance in simulated activities of daily living in glaucoma patients. Journal of glaucoma. 2020 Oct 30;29(10):970-4. | No outcome of interest |
| 2 | Christ SL, Zheng DD, Swenor BK, Lam BL, West SK, Tannenbaum SL, Muñoz BE, Lee DJ. Longitudinal relationships among visual acuity, daily functional status, and mortality: the Salisbury Eye Evaluation Study. JAMA ophthalmology. 2014 Dec 1;132(12):1400-6. | No outcome of interest |
| 3 | Dahlin-Ivanoff S, Sonn U. Use of assistive devices in daily activities among 85-year-olds living at home focusing especially on the visually impaired. Disability and Rehabilitation. 2004 Jan 1;26(24):1423-30. | No outcome of interest |
| 4 | Dunlop DD, Manheim LM, Sohn MW, Liu X, Chang RW. Incidence of functional limitation in older adults: the impact of gender, race, and chronic conditions. Archives of physical medicine and rehabilitation. 2002 Jul 1;83(7):964-71. | No outcome of interest |
| 5 | Duquette J, Loiselle J, Fréchette C, Déry L, Senécal MJ. Occupational performance in the basic and instrumental daily activities of persons with low vision who received rehabilitation services. British Journal of Occupational Therapy. 2019 Aug;82(8):457-65. | No outcome of interest |
| 6 | Griffith L, Raina P, Wu H, Zhu B, Stathokostas L. Population attributable risk for functional disability associated with chronic conditions in Canadian older adults. Age and ageing. 2010 Nov 1;39(6):738-45. | No outcome of interest |
| 7 | Guthrie DM, Declercq A, Finne-Soveri H, Fries BE, Hirdes JP. The health and well-being of older adults with dual sensory impairment (DSI) in four countries. PloS one. 2016 May 5;11(5):e0155073. | No outcome of interest |
| 8 | Haanes GG, Kirkevold M, Horgen G, Hofoss D, Eilertsen G. Sensory impairments in community health care: a descriptive study of hearing and vision among elderly Norwegians living at home. Journal of multidisciplinary healthcare. 2014 May 28:217-25. | No outcome of interest |
| 9 | Ishihara* K, Ishihara S, Nagamachi M, Osaki H, Hiramatsu S. Independence of older adults in performing instrumental activities of daily living (IADLs) and the relation of this performance to visual abilities. Theoretical Issues in Ergonomics Science. 2004 May 1;5(3):198-213. | No outcome of interest |
| 10 | Jones N, Bartlett HE, Cooke R. An analysis of the impact of visual impairment on activities of daily living and vision-related quality of life in a visually impaired adult population. British Journal of Visual Impairment. 2019 Jan;37(1):50-63. | No outcome of interest |
| 11 | Kempen GI, Ballemans J, Ranchor AV, van Rens GH, Zijlstra GR. The impact of low vision on activities of daily living, symptoms of depression, feelings of anxiety and social support in community-living older adults seeking vision rehabilitation services. Quality of life research. 2012 Oct;21:1405-11. | No outcome of interest |
| 12 | Klein BE, Moss SE, Klein R, Lee KE, Cruickshanks KJ. Associations of visual function with physical outcomes and limitations 5 years later in an older population: the Beaver Dam eye study. Ophthalmology. 2003 Apr 1;110(4):644-50. | No outcome of interest |
| 13 | Lamoureux EL, Hassell JB, Keeffe JE. The determinants of participation in activities of daily living in people with impaired vision. American journal of ophthalmology. 2004 Feb 1;137(2):265-70. | No outcome of interest |
| 14 | Lange R, Kumagai A, Weiss S, Zaffke KB, Day S, Wicker D, Howson A, Jayasundera KT, Smolinski L, Hedlich C, Lee PP. Vision-related quality of life in adults with severe peripheral vision loss: a qualitative interview study. Journal of Patient-Reported Outcomes. 2021 Dec;5:1-2. | No outcome of interest |
| 15 | Leroi I, Simkin Z, Hooper E, Wolski L, Abrams H, Armitage CJ, Camacho E, Charalambous AP, Collin F, Constantinidou F, Dawes P. Impact of an intervention to support hearing and vision in dementia: The SENSE‐Cog Field Trial. International journal of geriatric psychiatry. 2020 Apr;35(4):348-57. | No outcome of interest |
| 16 | Lino VT, Rodrigues NC, Andrade MK, Reis IN, Lopes LA, Atie S. Association between visual problems, insufficient emotional support and urinary incontinence with disability in elderly people living in a poor district in Rio de Janeiro, Brazil: A six-year follow-up study. PloS one. 2019 May 31;14(5):e0217456. | No outcome of interest |
| 17 | Mandas A, Mereu RM, Catte O, Saba A, Serchisu L, Costaggiu D, Peiretti E, Caminiti G, Vinci M, Casu M, Piludu S. Cognitive impairment and age-related vision disorders: their possible relationship and the evaluation of the use of aspirin and statins in a 65 years-and-over Sardinian population. Frontiers in Aging Neuroscience. 2014 Nov 7;6:309. | No outcome of interest |
| 18 | Rehab RO. Improving management of visually impaired patients from occupational therapy perspective: A case report. Med J Malaysia. 2021 Jul;76(4):579. | Case study |
| 19 | Odden JL, Mihailovic A, Boland MV, Friedman DS, West SK, Ramulu PY. Assessing functional disability in glaucoma: the relative importance of central versus far peripheral visual fields. Investigative Ophthalmology & Visual Science. 2020 Nov 2;61(13):23-. | No outcome of interest |
| 20 | Omar R, Rahman MH, Knight VF, Mustaphal M, Mohammed Z. Mental health state and quality of life questionnaire in low vision assessment: a case report. BMC research notes. 2014 Dec;7:1-4. | Case study |
| 21 | Patel R, Srivastava S, Kumar P, Chauhan S, Govindu MD, Jean Simon D. Socio-economic inequality in functional disability and impairments with focus on instrumental activity of daily living: a study on older adults in India. BMC public health. 2021 Dec;21:1-3. | No outcome of interest |
| 22 | Robertson N, Burden ML, Burden AC. Psychological morbidity and problems of daily living in people with visual loss and diabetes: do they differ from people without diabetes?. Diabetic medicine. 2006 Oct;23(10):1110-6. | No outcome of interest |
| 23 | Schilling OK, Wahl HW, Boerner K, Reinhardt JP, Brennan-Ing M, Horowitz A. Change in psychological control in visually impaired older adults over 2 years: Role of functional ability and depressed mood. Journals of Gerontology Series B: Psychological Sciences and Social Sciences. 2013 Sep 1;68(5):750-61. | No outcome of interest |
| 24 | Servat JJ, Risco M, Nakasato YR, Bernardino CR. Visual impairment in the elderly: impact on functional ability and quality of life. Clin Geriatr. 2011 Jul;19(7). | Review |
| 25 | Sgaramella TM, Nota L, Carrieri L, Soresi S, Sato G. Daily functioning, problem solving and satisfaction for quality of life in visually impaired old persons. International Journal on Disability and Human Development. 2017 Jan 20;16(2):225-32. | No outcome of interest |
| 26 | Singh RR, Maurya P. Visual impairment and falls among older adults and elderly: evidence from longitudinal study of ageing in India. BMC public health. 2022 Dec;22(1):1-1. | No outcome of interest |
| 27 | Sloan FA, Picone G, Brown DS, Lee PP. Longitudinal analysis of the relationship between regular eye examinations and changes in visual and functional status. Journal of the American Geriatrics Society. 2005 Nov;53(11):1867-74. | No outcome of interest |
| 28 | Soler V, Sourdet S, Balardy L, Abellan van Kan G, Brechemier D, Rouge Bugat ME, Tavassoli N, Cassagne M, Malecaze F, Nourhashemi F, Vellas B. Visual impairment screening at the Geriatric Frailty Clinic for Assessment of Frailty and Prevention of Disability at the Gérontopôle. The journal of nutrition, health & aging. 2016 Oct;20:870-7. | No outcome of interest |
| 29 | Soto‐Perez‐de‐Celis E, Sun CL, Tew WP, Mohile SG, Gajra A, Klepin HD, Owusu C, Gross CP, Muss HB, Lichtman SM, Chapman AE. Association between patient‐reported hearing and visual impairments and functional, psychological, and cognitive status among older adults with cancer. Cancer. 2018 Aug 1;124(15):3249-56. | No outcome of interest |
| 30 | Stevelink SA, Malcolm EM, Fear NT. Visual impairment, coping strategies and impact on daily life: a qualitative study among working-age UK ex-service personnel. BMC public health. 2015 Dec;15(1):1-7. | No outcome of interest |
| 31 | Wahl HW, Heyl V, Schilling O. The role of vision impairment for the outdoor activity and life satisfaction of older adults: A multi-faceted view. Visual Impairment Research. 2002 Jan 1;4(3):143-60. | No outcome of interest |
| 32 | Wahl HW, Heyl V, Schilling O. The role of vision impairment for the outdoor activity and life satisfaction of older adults: A multi-faceted view. Visual Impairment Research. 2002 Jan 1;4(3):143-60. | No outcome of interest |
| 33 | Wahl HW, Heyl V, Schilling O. The role of vision impairment for the outdoor activity and life satisfaction of older adults: A multi-faceted view. Visual Impairment Research. 2002 Jan 1;4(3):143-60. | No outcome of interest |
| 34 | Wood J, S Rubin G, Owsley C. The role of vision in everyday activities. Ophthalmic and Physiological Optics. 2011 May;31(3):201-2. | Review |
| 35 | Zheng DD, Christ SL, Lam BL, Tannenbaum SL, Bokman CL, Arheart KL, McClure LA, Fernandez CA, Lee DJ. Visual acuity and increased mortality: the role of allostatic load and functional status. Investigative ophthalmology & visual science. 2014 Aug 1;55(8):5144-50. | No outcome of interest |
