# Supplementary material for: Vision impairment and associated daily activity limitation: A systematic review and meta-analysis
Source: PLoS One. 2025 Jan 31;20(1):e0317452. doi: 10.1371/journal.pone.0317452 (PMC11785307; doi:10.1371/journal.pone.0317452)
Supplement: S4 Table — (DOCX) [file pone.0317452.s006.docx]

# **Supplementary Table 4.** Quality assessment and publication bias evaluation of included study using the Newcastle-Ottawa Scale (NOS)

| **Cohort study** | **Selection (4)** | | | | **Comparability (2)** | | **Outcome (3)** | | | **Total** |
| --- | --- | --- | --- | --- | --- | --- | --- | --- | --- | --- |
| **Author** | **Representativeness of exposed cohort** | **Selection** | **Ascertainment of exposure** | **Objectively measured vision impairment** | **Objectively measured ADL/IADL** | **Control of confounding factors** | **Assessment of vision impairment characteristics** | **Was follow-up long enough for outcomes to occur** | **Adequacy of follow up of cohorts** |  |
| Bekibele et al. 2008 | 1 | 1 | 1 | 0 | 0 | 1 | 1 | 1 | 1 | 7 |
| Bouscaren et al. 2019 | 1 | 1 | 1 | 0 | 0 | 1 | 0 | 1 | 1 | 6 |
| Brennan et al. 2005 | 1 | 1 | 1 | 0 | 0 | 1 | 0 | 1 | 1 | 6 |
| Cao et al. 2021 | 1 | 1 | 1 | 1 | 0 | 1 | 0 | 1 | 1 | 7 |
| Crews et al. 2004 | 1 | 1 | 1 | 0 | 0 | 1 | 0 | 1 | 1 | 6 |
| Dargent-Molinaet al. 1996 | 1 | 1 | 1 | 0 | 0 | 1 | 1 | 1 | 1 | 7 |
| Grue et al. 2009 | 1 | 1 | 1 | 0 | 0 | 1 | 0 | 1 | 1 | 6 |
| Horowitz et al. 1994 | 1 | 1 | 1 | 0 | 0 | 1 | 0 | 1 | 1 | 6 |
| Lam et al. 2013 | 1 | 1 | 1 | 0 | 0 | 1 | 0 | 1 | 1 | 6 |
| Liu et al. 2016 | 1 | 1 | 1 | 0 | 0 | 1 | 0 | 1 | 1 | 6 |
| Mueller-Schotte et al. 2019 | 1 | 1 | 1 | 0 | 0 | 1 | 0 | 1 | 1 | 6 |
| Pér`es et al. 2017 | 1 | 1 | 1 | 1 | 1 | 1 | 1 | 1 | 1 | 9 |
| Reuben et al. 1999 | 1 | 1 | 1 | 1 | 1 | 1 | 1 | 1 | 1 | 9 |
| Swanson et al. 2004 | 1 | 1 | 1 | 0 | 0 | 1 | 0 | 1 | 1 | 6 |
| Rovner et al. 1998 | 1 | 1 | 1 | 0 | 0 | 1 | 0 | 1 | 1 | 6 |
| Tareque et al. 2019 | 1 | 1 | 1 | 0 | 0 | 1 | 0 | 1 | 1 | 6 |
| Verbeek et al. 2022 | 1 | 1 | 1 | 0 | 0 | 0 | 1 | 1 | 1 | 6 |
| Wahl et al. 1999 | 1 | 1 | 1 | 0 | 0 | 1 | 1 | 1 | 1 | 7 |
| Wallhagen et al. 2001 | 1 | 1 | 1 | 0 | 0 | 1 | 0 | 1 | 1 | 6 |
| West et al. 1997 | 1 | 1 | 1 | 1 | 0 | 1 | 1 | 1 | 1 | 8 |
| Whitson et al. 2007 | 1 | 1 | 1 | 0 | 0 | 1 | 0 | 1 | 1 | 6 |
| Zhang et al. 2022 | 1 | 1 | 1 | 0 | 0 | 1 | 0 | 1 | 1 | 6 |
| **Cross-sectional study** | **Selection (5)** | | | | **Comparability Confounding factors are controlled (2)** | | **Outcome (3)** | | | **Total** |
| **Author** | **Representativeness of the sample** | **Sample size** | **Selection** | **Ascertainment of exposure** | **The subjects in different outcome groups are comparable, based on the study design or analysis.** | | **Objectively assessment of the outcomes** | | **Statistical test** |  |
| Berger et al. 2008 | 1 | 1 | 1 | 0 | 2 | | 0 | | 1 | 6 |
| Cacciatore et al. 2004 | 1 | 1 | 1 | 0 | 1 | | 0 | | 1 | 5 |
| Cahn et al. 2021 | 1 | 1 | 1 | 0 | 1 | | 0 | | 1 | 5 |
| Cimarolli et al. 2014 | 1 | 1 | 1 | 0 | 1 | | 0 | | 1 | 5 |
| Daien et al. 2014 | 1 | 1 | 1 | 1 | 1 | | 0 | | 1 | 6 |
| Dijkhuizen et al. 2016 | 1 | 1 | 1 | 0 | 1 | | 0 | | 1 | 5 |
| Ensrud et al. 1994 | 1 | 1 | 1 | 0 | 2 | | 1 | | 1 | 7 |
| Falahaty et al. 2015 | 1 | 1 | 1 | 1 | 1 | | 0 | | 1 | 6 |
| Guo et al. 2021 | 1 | 1 | 1 | 1 | 1 | | 1 | | 1 | 7 |
| Guthrie et al. 2018 | 1 | 1 | 1 | 0 | 1 | | 1 | | 1 | 6 |
| Harada et al. 2008 | 1 | 1 | 1 | 1 | 1 | | 0 | | 1 | 6 |
| Haymes et al. 2002 | 1 | 1 | 1 | 1 | 1 | | 0 | | 1 | 5 |
| Hochberget al. 2012 | 1 | 1 | 1 | 1 | 1 | | 0 | | 1 | 6 |
| Ivanoff et al. 2000 | 1 | 1 | 1 | 2 | 1 | | 1 | | 1 | 8 |
| Kee et al. 2021 | 1 | 1 | 1 | 1 | 1 | | 0 | | 1 | 6 |
| Keller et al. 1999 | 1 | 1 | 1 | 1 | 1 | | 0 | | 1 | 6 |
| Laitinen et al. 2007 | 1 | 1 | 1 | 1 | 1 | | 1 | | 1 | 7 |
| Mercan et al. 2021 | 1 | 1 | 1 | 0 | 1 | | 0 | | 1 | 5 |
| Naël et al. 2017 | 1 | 1 | 1 | 2 | 1 | | 1 | | 1 | 8 |
| Park et al. 2015 | 1 | 1 | 1 | 0 | 1 | | 1 | | 1 | 6 |
| Qiu et al. 2014 | 1 | 1 | 1 | 0 | 1 | | 1 | | 1 | 6 |
| Rokicki et al. 2016 | 1 | 1 | 1 | 1 | 1 | | 0 | | 1 | 6 |
| Ross et al. 1991 | 1 | 1 | 1 | 0 | 1 | | 0 | | 1 | 5 |
| Rubin et al. 1994 | 1 | 1 | 1 | 0 | 1 | | 1 | | 1 | 6 |

#

# 
