# Supplementary material for: Vision impairment and associated daily activity limitation: A systematic review and meta-analysis
Source: PLoS One. 2025 Jan 31;20(1):e0317452. doi: 10.1371/journal.pone.0317452 (PMC11785307; doi:10.1371/journal.pone.0317452)
Supplement: S1 Fig — (DOCX) [file pone.0317452.s007.docx]

# **Supplementary Figure 1a.** Funnel plot for publication bias: ADL difficulties for people with 18-99 years

*Egger’s* test P-value: 0.74; *Begg’s* test P-value: 0.49

# **Supplementary Figure 1b.** Funnel plot for publication bias: ADL difficulties for people with ≥65 years

*Egger’s* test P-value: 0.50; *Begg’s* test P-value: 0.19

# **Supplementary Figure 1c.** Funnel plot for publication bias: ADL difficulties for moderate to severe vision impairment.

*Egger’s* test P-value: 0.90; *Begg’s* test P-value: 0.42

# **Supplementary Figure 1d.** Funnel plot for publication bias: Self-reported vision impairment and ADL difficulty

*Egger’s* test P-value: 0.90; *Begg’s* test P-value: 0.49

# **Supplementary Figure 1e.** Funnel plot for publication bias: Objectively measured vision impairment and ADL difficulty

*Egger’s* test P-value: 0.51; *Begg’s* test P-value: 0.37

# **Supplementary Figure 1f.** Funnel plot for publication bias: Self-reported ADL difficulty and vision impairment

*Egger’s* test P-value: 0.90; *Begg’s* test P-value: 0.42

# **Supplementary Figure 1g.** Funnel plot for publication bias: Objectively-measurement ADL difficulty and vision impairment

*Egger’s* test P-value: 0.77; *Begg’s* test P-value: 1.00

# **Supplementary Figure 1h.** Funnel plot for publication bias: IADL difficulties in people with 18-99 years

*Egger’s* test P-value: 0.89; *Begg’s* test P-value: 0.39

# **Supplementary Figure 1i.** Funnel plot for publication bias: ADL difficulties IADL difficulties in people with ≥65 years

*Egger’s* test P-value: 0.78; *Begg’s* test P-value: 0.21

# **Supplementary Figure 1j.** Funnel plot for publication bias: Self-reported IADL difficulty and vision impairment

*Egger’s* test P-value: 0.57; *Begg’s* test P-value: 0.28

# **Supplementary Figure 1k.** Funnel plot for publication bias: Objectively measured IADL difficulty and vision impairment

*Egger’s* test P-value: 0.59; *Begg’s* test P-value: 0.19

# **Supplementary Figure 1l.** Funnel plot for publication bias: IADL difficulties for moderate to severe vision impairment

*Egger’s* test P-value: 0.81; *Begg’s* test P-value: 0.55

# **Supplementary Figure 1m.** Funnel plot for publication bias: Objectively measured vision impairment and IADL difficulty

*Egger’s* test P-value: 0.60; *Begg’s* test P-value: 0.52

# **Supplementary Figure 1n.** Funnel plot for publication bias: Self-reported vision impairment and ADL difficulty

*Egger’s* test P-value: 0.44; *Begg’s* test P-value: 0.24
