# Supplementary material for: Vision impairment and associated daily activity limitation: A systematic review and meta-analysis
Source: PLoS One. 2025 Jan 31;20(1):e0317452. doi: 10.1371/journal.pone.0317452 (PMC11785307; doi:10.1371/journal.pone.0317452)
Supplement: S2 Fig — (DOCX) [file pone.0317452.s008.docx]

# **Supplementary Figure 2.** Meta-regression analysis for the association between vision impairment and difficulties with activity of daily living based on age.
