# Supplementary material for: Vision impairment and associated daily activity limitation: A systematic review and meta-analysis
Source: PLoS One. 2025 Jan 31;20(1):e0317452. doi: 10.1371/journal.pone.0317452 (PMC11785307; doi:10.1371/journal.pone.0317452)
Supplement: Attachment — Submitted filename: review.pdf [file pone.0317452.s011.pdf]

Summary: The paper reports the results of a meta-analysis of 46 studies involving 210,960 participants on the relationship between vision impairment and measures of difficulties in daily life. The paper focuses on two broad measures: 1) basic activities of daily living (ADL), which refer to fundamental skills for daily self-care like eating and bathing, and 2) instrumental ADL, which refers to more complex activities like managing finances and preparing meals. The meta-analysis consisted of random effects models to calculate the pooled effect size for the correlation between vision impairment and the target measures. The results were a large positive correlation between vision impairment and difficulties in daily life, which suggests that worsening vision results in greater difficulties in daily life. Although the results of this study are correlational, the meta-analytic approach strengthens the claim that the relationship between vision impairment and difficulties with daily life is real and that causal factors and interventions should be investigated.

Opinion: This paper is a systematic review. The methods ensure a comprehensive and unbiased sampling of the existing literature, and the results have not been published elsewhere. The article adheres to appropriate reporting guidelines and standards for data availability. The article is presented in an intelligible fashion and the conclusions are clearly presented and are supported by the data. Most of the methods/statistics are performed to a high technical standard and described in sufficient detail. However, my main concern is about the age variable and specifically how the authors attempted to evaluate the role of age using the subgroups analysis (see review). Age is likely a mediator between visual impairment and difficulty in daily life, and the current approach taken by the authors does not adequately rule this out this mediating relationship. If this analysis is included and the current results stand, then this paper should be published.

Major:

1. I am particularly interested in the age variable. I expect that older individuals and those with disabilities have much higher scores on basic ADL, regardless of whether impaired vision is their disability. Nonetheless, vision often gets worse with age which means that age may be a mediating variable. The subgroup analysis (pp 279-307) on age attempts to address this issue, but the approach was quite odd. The authors compared the pooled effect in individuals aged 18-99 (presumably the entire sample) but also in individuals aged 65 year or more, and found no difference between the groups. Based on the reporting, it appears that the first group contains the members of the second group. At the very least, this needs to be better explained and justified. I would like to see a true subgroup analysis comparing different age groups. The best approach, however, would be for age to be included as a factor in the random effects model.

Minor:

1. Consider addition of summary stats in results. Basic ADL describes activities associated with basic physical needs like eating or toileting, whereas instrumental ADL involves more complicated cognitive tasks, such as balancing a checkbook or following a recipe. These two measures are very different in that individuals who have high difficulty with basic ADL are unable, or nearly unable, to physically care for themselves at all. In contrast, for IADL someone could have high difficulties with certain tasks like meal preparation but be entirely able to care for themselves. I think it is important for the authors to include some data about the range of these scores, and how these scores themselves relate to each other and

to other variables such as age. This would give insight into the general abilities of the subjects without considering visual ability.

2. Clarify lines 283-288: Subgroup analysis based on the different assessments of vision impairment showed a higher non-significant difficulty in IADL. What is “non-significant difficulty”? In the following sentence you say “significant difficulty”. Non-significant versus significant difficulties need to be better defined, and the term significant should be avoided here where you are also using the same term to describe the results of significance tests.
3. Edit awkward/extraneous lines 330-333: The ROC results from another study should not be reported in the Discussion, and the way the ROC results are reported is vague and uninformative. I suggest complete removal of the ROC results and a higher-level description of the relationship between visual acuity threshold and IADL.
4. Explain lines 352-355: Statistical heterogeneity does not increase just because there is a large number of studies in the meta-analysis, but rather that there were variations in the original study designs such that there is more random error than just measurement error. This is obvious from the details in Table 1. The authors somewhat address this issue by performing a subgroup analysis on the different measures that make up the broad ADL and IADL category measures. However, it would be helpful for the authors to spend a bit more time discussing these differences rather than brushing it off as “expected given the large number of studies”.
