# Supplementary material for: Vision impairment and associated daily activity limitation: A systematic review and meta-analysis
Source: PLoS One. 2025 Jan 31;20(1):e0317452. doi: 10.1371/journal.pone.0317452 (PMC11785307; doi:10.1371/journal.pone.0317452)
Supplement: Attachment — Submitted filename: PONE-D-24-38439_reviewr comments.docx [file pone.0317452.s010.docx]

PONE-D-24-38439_Review

Vision impairment and associated daily activity limitation: A systematic review 2 and meta-analysis

Generally a well thought, well written manuscript.

Abstract

Background: Doesn’t reflect scholarly inquest or bring out the problem that the researchers sought to study.

Review the grammar of sentence starting on line 38-40 to make it clear

Review standard P- value reporting for values less than 0.0001 (see line 41)

General comment: Tables and figures should have been included in the document for ease of corroboration of the figures, then if the document proceeds to productions then can be added and Figures, tables and supporting documents in the required file versions

L230, 275: review grammar. Sentence should not start with number symbol.

Authors to review publication policy on supporting information, supplemental material and figures and tables with data.

Some of the references are too old e.g ref 5, 6
